# Identification of a Distinct Small Cell Population from Human Bone Marrow Reveals Its Multipotency *In Vivo* and *In Vitro*

**DOI:** 10.1371/journal.pone.0085112

**Published:** 2014-01-17

**Authors:** James Wang, Xiaoyu Guo, Monica Lui, Pei-Ju Chu, Jennifer Yoo, Megan Chang, Yun Yen

**Affiliations:** 1 StemBios Technologies, Inc., Monterey Park, California, United States of America; 2 Board Member of the Scientific Advisory Board, StemBios Technologies, Inc., Monterey Park, California, United States of America; University of Miami School of Medicine, United States of America

## Abstract

Small stem cells, such as spore-like cells, blastomere-like stem cells (BLSCs), and very-small embryonic-like stem cells (VSELs) have been described in recent studies, although their multipotency in human tissues has not yet been confirmed. Here, we report the discovery of adult multipotent stem cells derived from human bone marrow, which we call **S**tem**B**ios (SB) cells. These isolated SB cells are smaller than 6 ìm and are DAPI+ and Lgr5+ (Leucine-Rich Repeat Containing G Protein-Coupled Receptor 5). Because Lgr5 has been characterized as a stem cell marker in the intestine, we hypothesized that SB cells may have a similar function. *In vivo* cell tracking assays confirmed that SB cells give rise to three types of cells, and *in vitro* studies demonstrated that SB cells cultured in proprietary media are able to grow to 6–25 ìm in size. Once the SB cells have attached to the wells, they differentiate into different cell lineages upon exposure to specific differentiation media. We are the first to demonstrate that stem cells smaller than 6 ìm can differentiate both *in vivo* and *in vitro*. In the future, we hope that SB cells will be used therapeutically to cure degenerative diseases.

## Introduction

Stem cells have been derived from the inner cell mass (hESCs: human Embryonic Stem Cells), adult tissues [1]–[4], and adult somatic cells (iPSs: induced Pluripotent Stem Cells) [5], [6]. Although hESCs and iPSs are capable of becoming almost any type of specialized cell in the body and may have the potential to generate replacement cells for a broad array of tissues and organs [7], future transplantations using these cells are hindered by immune rejection and teratoma formation [8]–[12]. As a result, the search for multipotent or pluripotent stem cells from adult tissue has become increasingly important in stem cell research. Adult stem cells, unlike hESCs and iPSs, do not undergo teratoma formation [13], [14] or immune rejection in autologous transplants.

Currently, adult stem cell studies have focused on MSCs (mesenchymal stem cells) [15], which are considered a promising therapeutic approach for many diseases [16]–[18]. They can be isolated from sources such as umbilical cord blood [19] and bone marrow [20], among others [21], [22]. However, plasticity of MSCs for trans-differentiation is questionable because they appear to differentiate into mesoderm lineages only [15]. Thus, obtaining multipotent or pluripotent adult stem cells that can differentiate into different germ layers is crucial for future cell therapy.

The race to discover adult pluripotent stem cells began early this century. In 2002, Jiang et al. [23] reported their findings on multipotent adult progenitor cells (MAPCs) from bone marrow that could differentiate into ectoderm, mesoderm, and endoderm cells, introducing a new avenue in the discovery of pluripotent or multipotent stem cells from adult tissue. Other types of cells including marrow-isolated adult multi-lineage inducible cells (MIAMI) [24] and single cell clones derived from bone marrow [25] demonstrated the same multi-potential ability for differentiation. Unfortunately, the difficulty associated with obtaining, culturing, and expanding these pluripotent stem cells has proven to be a challenge.

In this study, we present our discovery of one group of novel cells isolated from human Bone Marrow (hBM). These cells, which we call SB cells, are less than 6 µm in diameter, express Lgr5, and experience significant increases in size and population after incubation *in vitro*. The addition of specific differentiation media at this stage caused the SB cells to differentiate into endoderm-, mesoderm-, and ectoderm-derived cell types. *In vivo* tracking of SB cells that were intravenously injected into the tails of sub-lethally irradiated SCID mice showed that the SB cells were able to develop into hepatocytes (endoderm), neurons (ectoderm), and skeletal muscle cells (mesoderm). Overall, these characteristics suggested that SB cells could play large roles in future stem cell-based therapeutic applications.

## Materials and Methods

### 1.1. Ethics Statement

All mouse injections and organ preparations were carried out at Charles River Laboratories (protocol numbers: BA-p042 and BA-e219) in accordance with the recommendations in the Guide for the Care and Use of Laboratory Animals of the National Institutes of Health and the Animal Welfare Act. The protocol was approved by the Institutional Animal Care and Use Committee of Charles River Discovery Research Services in North Carolina (permit number: 990202).

### 1.2. Samples and reagents

This study was conducted using data obtained from 70 fresh hPB and 30 fresh hBM samples purchased from AllCells, LLC. A list of the reagents and antibodies used in this study is available upon request. The antibodies used for flow cytometry are as follows: SYTO Green nucleic acid staining (Life Technologies), CD9 (Biolegend), CD235a (eBioscience), Lgr5 (Origene), Lin (BD), CD45 (BioLegend), CD34 (eBioscience), CXCR4 (eBioscience), CD117 (eBioscience), CD105 (eBioscience), CD133 (Miltenyi Biotech) and CD66e (Santa Cruz Biotechnology). A custom Y-chromosome FISH probe (Empire Genomics) was used for FISH staining.

### 1.3. Isolation of the SB mixture

hBM and hPB were collected in anti-clotting tubes and incubated at 4°C for 72 hours, after which the blood and bone marrow were separated into two layers. The SB mixture was collected from the top layer.

### 1.4. Isolation of Lgr5+ cells from the SB mixture

The Lgr5+ cells were isolated using two methods: the magnetic enrichment of Lgr5+ cells (performed seven times) and FACSorting (performed five times). The PE Selection Kit (StemCell Technologies, catalog number: 18551) was used to isolate the Lgr5+ cells. The SB mixture was incubated with a PE selection cocktail (using an Lgr5-PE antibody) for 15 minutes and magnetic nanoparticles for 10 minutes at room temperature (RT). The mixture was placed into the magnet and incubated for 5 minutes at RT. The supernatant was then discarded, and the cells were plated for further culturing. Alternatively, the cells of the SB mixture were stained with the Lgr5-PE antibody and isolated via FACSorting using the BD FACSAria cell sorter at the UCLA Flow Cytometry Core Facility.

### 1.5. SB cell cultures

Purified SB cells were plated onto a collagen-coated 6-well plate (Thermo Scientific, catalog number: 152034) in SB medium and monitored daily until the cells attached to the well. The SB cell suspension was cultured in Stem Pro 34 medium (Life Technologies) with 1X antibiotic, 1X L-glutamine, 5 ng/mL G-CSF, 5 ng/mL SCF, 40 ng/mL EGF, 20 ng/mL bFGF, 5 ng/mL PDGF, 10 ng/mL R-spondin-1, and 10 ng/mL Noggin to allow for cell expansion and enlargement to a size of 6∼25 µm for several days until cell attachment. After the cells attached, they were cultured in Mesengro MSC medium (Stem RD) and were ready for differentiation. For SB cell induction, please refer to the differentiation assay described below.

### 1.6. Doubling Time and Cell Cycle Assay

For the doubling time assay, purified Lgr5+ cells and Lgr5- cells were plated in 48-well plates at 3×10^4^ cells/well. A volume of 200 µl medium was added to each well (10 ng/ml GCSF (eBioscience), 10 ng/ml SCF (Peprotech), 10 ng/ml EGF (Peprotech), 10 ng/ml R-spondin-1 (StemRD), PDGF (eBioscience), and Opti-MEM Reduced-Serum Medium (Invitrogen)). The total number of cells in each well was counted in triplicate using a hemocytometer at 0 hrs, 24 hrs, 48 hrs, and 96 hrs of incubation. For the cell cycle assay, purified Lgr5+ cells were starved in 1% BSA/PBS solution for 16 hrs at 4°C. The Lgr5+ cells were plated in the same media as the doubling time assay and fixed in 70% ethanol at specific time points of 0 hrs, 8 hrs and 14 hrs. The cells were centrifuged at 6000 rpm for 15 minutes and the ethanol was removed. The cells were then stained with propidium iodide (PI) for DNA content and incubated for 5–10 minutes at room temperature. After staining, the sample was subjected to flow cytometry and the data were analyzed with ModFit software.

### 1.7. *In vivo* study: injection of SB cells into mice

SB cells from the BM of a 25-year-old, male human donor were purified and re-suspended in PBS with 5% human albumin for injection at Charles River Laboratories. Two groups of six female SCID mice (6–8 weeks old) received a sub-lethal (2Gγ) gamma-irradiation prior to injection. Each mouse was injected twice with 1×10^5^ SB cells, first immediately after the radiation and again 24 hrs later. The negative control group consisted of mice injected with PBS only. Tissues were collected 60 days after the first injection. Half of the tissues were prepared as frozen sections by Charles River Laboratories, and the remaining tissues were sent to StemBios in RNAlater reagent for gene expression analysis at the RNA level.

### 1.8. DAPI staining

The cells were smeared onto slides and incubated with 100% EtOH for 20 minutes at −20°C and then with DAPI for 30 minutes at RT. The slides were then washed twice with PBS and mounted with Vectashield mounting media (Vector, catalog number: H-1200).

### 1.9. Differentiation assay

SB cells were cultured in differentiation medium, which was changed every 2–3 days. As previously described [10], for hepatocyte differentiation, the cells were cultured in three different types of media: high glucose DMEM with 3% horse serum, 1X antibiotic, 1X L-glutamine, and 5 ng/mL activin for 4 days; high glucose DMEM with 3% horse serum, 1X antibiotic, 1X L-glutamine, 20 ng/mL bFGF, and 5 ng/mL hBMP2 for 10 days; and Hepato ZYME SFM (Life Technologies Gibco) with 2% horse serum, 1X antibiotic, 10 ng/mL HGF, 1×10 nM Dex, and OSM 10 ng/mL for 10 to 15 days. For neurogenic and adipogenic differentiation, the cells were grown in neuronal differentiation medium (Promocell, catalog number: C-28015) for 8–10 days and adipocytic differentiation medium (Life Technologies, catalog number: A10070-01) for 10 days, respectively, in accordance with the manufacturer's protocols. Adipocyte detection kits were used for adipocyte staining and detection.

### 1.10. Transwell test

Five-µm transwell plates (Corning, catalog number: 3421) were used for the co-culture experiments. Female stromal cells were plated at the bottom of the wells, and male SB cells were seeded on top. The cells were cultured in stromal cell medium (Life Technologies, catalog number: A1033201). After two days of incubation, the cells at the bottom of the wells were assayed by FISH staining.

### 1.11. Flow cytometry

Cells from the 70 hPB and 30 hBM samples were stained using the following antibodies: CD235a-APC, CD9-FITC, Lgr5-PE, CD133-APC, CD45-APC, CD34-APC, CXCR4-APC, CD117-APC, CD105-APC, Lin-FITC and SYTO-FITC. The cells (1×10^5^) were re-suspended in PBS containing 1% BSA (staining buffer) and incubated with the indicated antibodies (or labeled isotype control antibodies) for 30 min at 4°C. The cells were washed in flow cytometry staining buffer and analyzed with a C6 accuri flow cytometer.

### 1.12. ELISA

A human albumin ELISA kit (Bethyl Laboratories, catalog number: E88-129) was used to test the amount of albumin secretion from the cells in culture. The samples and standards were loaded in 96-well plates according to the manufacturer's instructions and were measured at an absorbance of 450 nm.

### 1.13. FISH

SB cells grown *in vitro* were first smeared onto Fisherbrand Colorfrost microscope slides and fixed in −20°C MeOH/CH_3_COOH (3∶1) for 1 hour at RT. The tissue section slides were prepared by Charles River Laboratories. All of the slides were washed in 2X SSC, pretreated with pepsin at 37°C, fixed with 1% formaldehyde in 0.5% MgCl_2_/PBS, and dehydrated with serial concentrations of ethanol. Fluorescence human Y chromosome probes (Empire Genomics) were loaded onto the slides and denatured at 80°C. The slides were then incubated overnight at 37°C and washed with 0.4X SSC/0.3% IGEPAL at 37°C and 2X SSC/0.1% IGEPAL at RT for 2 minutes each. The slides were then counterstained with DAPI (Vector Vectashield) and observed under a Nikon Ti-S fluorescence microscope.

### 1.14. RT-PCR for gene expression analysis

RNA was extracted from the SB cells using the Arcturus PicoPure RNA isolation kit from Life Technologies (catalog number: 12204-01); the extraction was followed by multiple steps of reverse transcription using the RiboAmp HS PLUS kit from Life Technologies (catalog number: KIT0525) and real-time PCR to detect Oct 4 and Nanog gene expression. For mouse tissue, RNA was extracted with the Qiagen RNA extraction kit (catalog number: 74104), reverse transcription was performed using the reverse transcription kit from Life Technologies (catalog number: 18080-051), and real-time PCR was performed using SYBR green mix from BioRad (catalog number: 170-8882) according to the manufacturer's instructions. The PCR products were analyzed on a 1.8% agarose gel. The PCR products that produced bands were sequenced to confirm that they corresponded to the genes of interest. The primer information is shown below:

GAPDH (F: 5′-AGCTGAACGGGAAGCTCACT-3′, R: 5′-TGCTGTAGCCAAATTCGTTG-3′)

mouse-specific β actin (F: 5′-AAGAGCTATGAGCTGCCTGA-3′, R: 5′-TACGGATGTCAACGTCACAC-3′)

α-1 anti-trypsin (F: 5′-GGGAAACTACAGCACCTGGA-3′, R: 5′-CCCCATTGCTGAAGACCTTA-3′)

human-specific Tau (F: 5′-CTCTTTCAGGGGTCCTAAGC-3′, R: 5′-AGCTGCAGGTCTGTAGATGG-3′)

human specific myogenic factor 4 (F: 5′-CAGTGCCATCCAGTACATCG-3′, R: 5′-AGGTTGTGGGCATCTGTAGG-3′)

alpha fetoprotein (F: 5′-AAATGCGTTTCTCGTTGCTT-3′, R: 5′-GCCACAGGCCAATAGTTTGT-3′)

albumin (F: 5′-GAAACATTCACCTTCCATGC-3′, R: 5′-ACAAAAGCTGCGAAATCATC-3′)

neurofilament (F: 5′-GCGTCTCCTCAGAAACAAAA-3′, R: 5′-GCACACAGGATAGAGGATGG-3′)

FoxA2 (F: 5′-CCATGCACTCGGCTTCCAGTATG-3′, R: 5′-CGCCGACATGCTCATGTACGTG-3′)

MAPT (F: 5′-CTCTTTCAGGGGTCCTAAGC-3′, R: 5′-AGCTGCAGGTCTGTAGATGG-3′)

Oct4 (F: 5′-GGACCAGTGTCCTTTCCTCT-3′, R: 5′-CCAGGTTTTCTTTCCCTAGC-3′)

Nanog (F: 5′–ATGAGTGTGGATCCAGCTTG–3′, R: 5′–CCTGAATAAGCAGATCCATGG-3′)

Sox2 (F: 5′-GAAATGGGAGGGGTGCAAAA-3′, R: 5′-ATCGCGGTTTTTGCGTGAGT-3′)

### 1.15. Immunohistochemistry

The frozen section slides were fixed in 3.7% formaldehyde, blocked with 10% normal horse serum from the ABC kit (VECTASTAIN Elite PK-6200; Vector), and incubated overnight with primary antibody at 4°C. The biotinylated universal secondary antibody (ABC Kit) complex method and the TSA Biotin system (Perkin Elmer, catalog number: NEL744001KT) were used for detection. In the final step, the slides were counter-stained with DAPI and observed under a Zeiss Upright LSM510 2-Photon confocal microscope.

### 1.16. Immunocytochemistry (ICC)

Frozen tissue section slides were fixed in 3.7% formaldehyde and blocked with 5% BSA in PBS. The slides were incubated with primary antibody overnight at 4°C and then secondary antibody for 1 hour in the dark at room temperature. For the final step, the slides were counterstained with DAPI and observed under a Nikon Ti-S fluorescence microscope.

### 1.17. Western Blot analysis

Cells were re-suspended in a RIPA buffer containing protease inhibitors (Thermo Scientific, catalog numbers: 78425 and 89900). The cell lysates were separated on 4–12% Bis-Tris gels (Life Technologies, catalog number: BG04120) and transferred to PVDF membranes (Life Technologies, catalog number: LC2005). After incubation with primary antibodies against beta-actin (Santa Cruz Biotech), Tau (Millipore), albumin (Abcam), and adiponectin (Abcam) at 4°C overnight, the membranes were incubated with an HRP-conjugated secondary antibody (1:50,000; Li-Cor) for 1 hour at room temperature. They were then incubated with WesternSure Premium Chemiluminescent Substrate (Li-Cor, catalog number: 926-95000) for 5 minutes and visualized using a Li-Cor C-Digit Blot Scanner.

### 1.18. Statistical analysis

T-tests were used to analyze the data from the adipogenesis assay and albumin ELISA. Statistical significance was defined as P<0.01.

## Results

### 2.1 SB cells are present in human bone marrow (hBM) and peripheral blood (hPB)

It has been suggested that small stem cells (smaller than 6 ìm in diameter), such as spore-like cells [26], [27], VSELs [28], [29], and BLSCs [30], may exist in all adult tissues. These cells primarily remain in a dormant state and may only be activated in harsh environments, such as in oxygen-deprived states and extreme temperature conditions. Injury or disease could also trigger activation, at which point these cells have the potential to regenerate into any tissue lineage. However, researchers have been skeptical regarding the characterization of these small stem cells as pluripotent or multipotent in humans [31]. Our aim was to obtain potentially useful multipotent or pluripotent stem cells from adult tissue, specifically from hBM or hPB.

**Figure 1:**
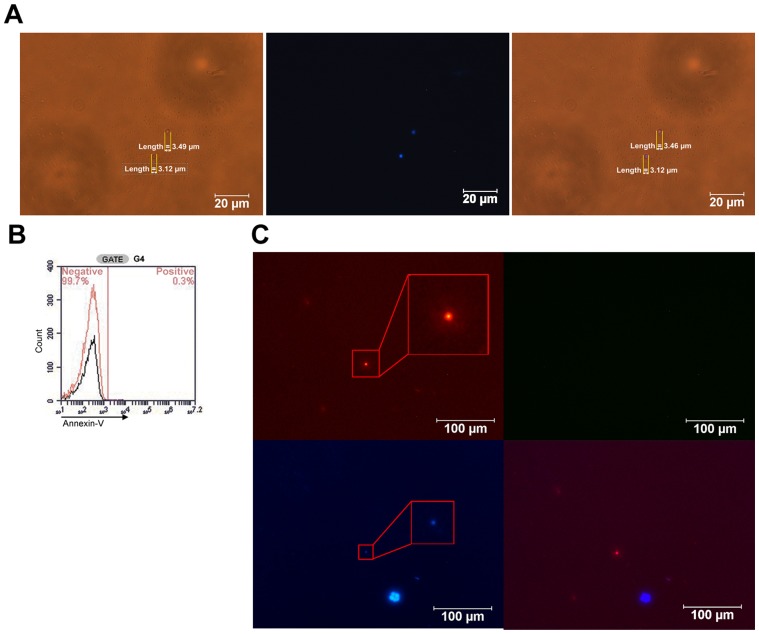
SB cells in hBM. **A) Presence of SB cells and DAPI staining.** The SB cells in the SB mixture layer were visualized under a light microscope. The cells of the SB mixture are shown under white light (left) and UV (middle). The merged image (right) reveals SB cells with a size of approximately 3.5 µm. The SB cells are the only nucleated cells in the SB mixture; thus, the SB cell count is equivalent to the number of DAPI+ cells. Scale bars, 20 µm. **B) Annexin-V staining.** The SB cells were analyzed for the expression of Annexin-V (black), a marker of apoptotic bodies, and for the isotype control (red). As shown, 99.7% of these cells did not express Annexin-V, indicating that these cells were not apoptotic bodies. **C) FISH and DAPI staining of SB cells in the 5-µm transwell assay.** Male SB cells were seeded at the top of the transwell, and stromal cells from female bone marrow were seeded at the bottom. FISH staining was performed immediately after the SB cells passed through the 5-µm filter. The SB and stromal cells were both DAPI-positive, but only the SB cells contained the Y-chromosome. Thus, the SB cells were double-positive for nuclear and Y-chromosome staining. One FISH-positive cell (top left) and two DAPI-positive cells (bottom left) are shown. The merged image (bottom right) reveals that the FISH-positive cell was also DAPI-positive, indicating that it is an SB cell. Scale bars, 100 µm.

**Figure 2:**
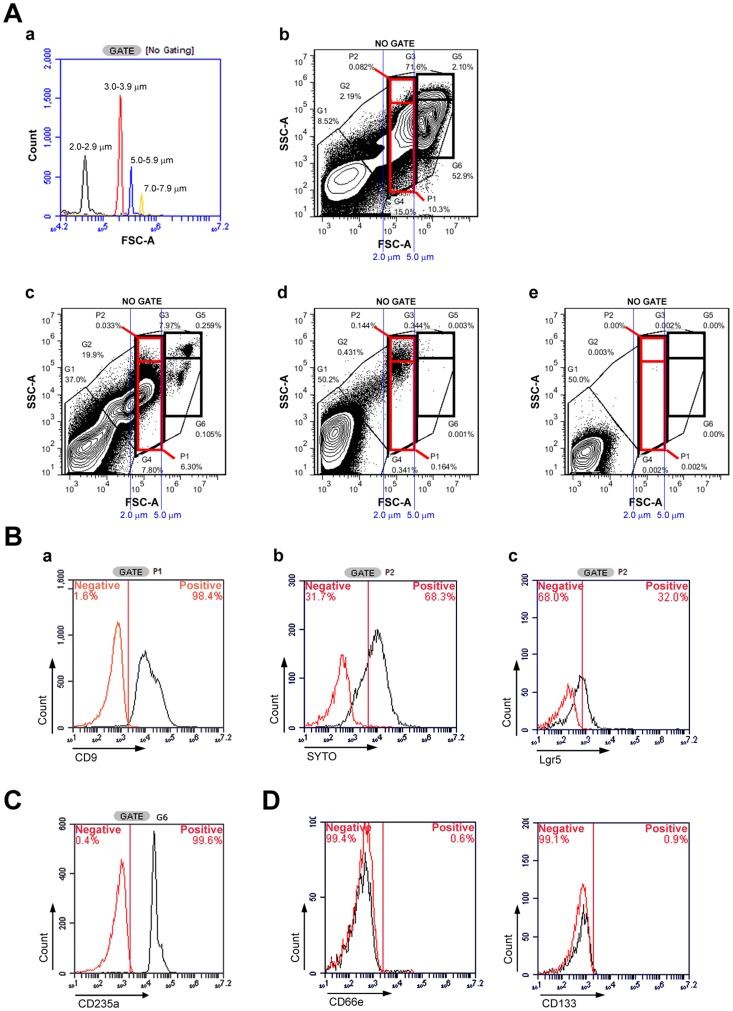
Distinct SB cell markers. **A) Flow cytometry of the SB mixture.** Beads were purchased from Spherotech, Inc. and examined by flow cytometry to determine the size range standards (a). Using these ranges as a reference, the SB cells were estimated to be 2–6 µm in diameter. Flow cytometry for the hPB and hBM samples before separation (b) and the layer containing only the SB mixture after separation (d). The addition of red blood cell (RBC) lysis buffer revealed that G6 contained RBCs (c). G1 gating represents the background of the staining buffer (e). G2 gating contained particles that were <1 µm in size; most of these were microparticles and microvesicles. G3 contained three major populations (>1 µm): SB cells (G4), RBCs (G6), and white blood cells (WBCs) (G5). The WBCs (G5) were larger than 6–7 µm and have nuclei. **B) Flow cytometry of the SB cells.** The G4 region was further divided into P1 and P2. (a) P1 gating represents the platelet population. Nearly all of the cells in this gate were CD9+. (b) P2 gating represents the SB cells. We found that 68.3% of these cells were SYTO+, indicating chromosome structure. (c) Lgr5, a stem cell marker, was expressed by 32% of the P2 population. Black: staining with Lgr5; red: staining with the isotype control. **C) Flow cytometry of RBCs in the SB cell mixture.** G6 represents the RBC location. The RBCs were CD235a+. **D) Flow Cytometry of VSELs and BLSCs in the SB cell mixture.** Few cells expressed CD66e, a marker of BLSCs (left) and CD133, a marker of VSELs (right), in the SB mixture.

hBM or hPB were collected in anti-clotting tubes and separated into two layers after 72 hours at 4°C. The bottom layer consisted almost entirely of red blood cells (RBC) and white blood cells, while the top layer, which we referred to as the SB mixture layer, contained SB cells (Fig. 1A). Because the SB cells resided in the top layer, the general size of the SB cells was estimated as >6 ìm, the size of the RBCs, which was further confirmed by the flow cytometry results in shown Fig. 2. This separation narrowed the identity of the cells in the SB mixture layer to SB cells, platelets, and extracellular vesicles including microparticles, microvesicles, and apoptotic bodies, because all of these cell types conform to this size restriction [32], [33]. Our results indicated that an average of less than 10% of the SB mixture cells were DAPI-positive; the DAPI-positive cells excluded platelets and extracellular vesicles, which lacked nuclei (Fig. 1C). To verify the presence of integral chromosome structures, we performed FISH Y-chromosome staining on the SB cells. The SB cells from the male donor were seeded at the top of the transwell, and the large stromal cells from the female donor were plated at the bottom. After 2 to 3 days of incubation, the SB cells were passed through a 5-ìm filter of the transwell, and the male SB cells were identified using Y-chromosome fluorescence. Around the large stromal cells, one small cell was identified that was positive for both FISH and DAPI staining (Fig. 1C), indicating that the SB cells contained an integral chromosome structure. Negative Annexin-V staining in these SB cells (Fig. 1B) further confirmed their identity by eliminating the possibility of apoptotic bodies. Thus, these cells were live SB cells and not apoptotic bodies, platelets or extracellular vesicles.

To further characterize the SB mixture, the cells were analyzed using flow cytometry. A total of 1,000,000 events were collected at a threshold of 10,000 in the CFlow software for each antibody-stained sample, and gates were created based on data from the 70 human Peripheral Blood (hPB) and 30 hBM samples. Flow cytometry size standard particles (Spherotech) were used to determine the size range of 2–6 µm (Fig. 2A-a) for gating analysis. Gate G2 was comprised of cells that were smaller than 2 µm, which were most likely microparticles or microvesicles. Gate G3, which included cells with a diameter greater than 2 µm, included three populations: G4, G5, and G6. Analysis using RBC lysis buffer indicated the presence of RBCs in the G6 region (Fig. 2A-c), where 99.6% of the cells in this region were confirmed as positive for CD235a (Fig. 2C) and negative for SYTO-stained nuclei (data not shown). The cells in the G5 region were also larger than RBCs (G6) and were presumed to be WBCs because these cells stained positive for CD11b (data not shown). The cells of the SB mixture were present in the G4 region, confirming that the SB cells were smaller than the RBCs (6 µm). The post-purification procedure removed nearly all of the RBCs (G6) and WBCs (G5) in the hPB and hBM samples (Fig. 2A-d), and staining with Lin antibodies excluded the lineage cells. In addition, more than 80% of the cells derived from hPB were positive for CD9, a platelet marker [34], in the G4 region; nearly all of these cells were captured in the P1 region (Fig. 2B-a). Approximately 10-20% of the G4 population did not express CD9, a platelet marker, and are shown in the P2 region. In this P2 region, 68.3% of the P2 region cells were positive for nuclear SYTO staining (Fig. 2B-b), and approximately 10%–60% of the cells (various individuals) expressed Lgr5 markers. These cells, which were both Lgr5+ and SYTO+, were named SB cells. In addition to existing in the peripheral blood, these SB cells were also found in the bone marrow. The SB mixture was also investigated for the presence of similar small stem cells, BLSCs and VSELs using the CD66e and CD133 markers, respectively. Less than 1% of the cells in the SB mixture expressed either CD66e or CD133 (Fig. 2D), suggesting that the VSEL and BLSC concentrations in this mixture were insignificant.

### 2.2 Characterization of Lgr5+ SB cells from hBM

Using magnetic beads or FACSorting, Lgr5+ SB cells were isolated from the SB mixture derived from BM. FACSorting was performed five times on five individual BM samples. Depending on each individual; approximately 0.3–3% of the SB mixture was composed of Lgr5+ cells. These Lgr5+ cells were further analyzed via DAPI staining. Positive DAPI expression was detected in approximately 70% of the Lgr5+ sorted cells; this result is detailed in Figure S1. A proliferation assay and cell cycle analysis were also performed on the Lgr5+ SB cells. The proliferation rate for the Lgr5+ cells was found to be 21.7 hours (Fig. S2A), and the cell cycle profile revealed that the proportions of cells in S and G2/M phase were larger than that in G1 phase (Fig. S2B). Both assays revealed characteristics that were similar to embryonic stem cells[35]. To further investigate whether our Lgr5+ cells had similar characteristics to embryonic stem cells, we examined their embryonic stem cell marker expression (Fig. S3). According to our RT-PCR gel results, our Lgr5+ cells expressed Nanog and Oct4, which are also expressed on embryonic stem cells, but did not express Sox2 markers (Fig. S3). Flow cytometry was used to compare these cells with HSCs, MSCs and VSELs. According to the hBM flow cytometry data, the Lgr5+ cells were negative for lineage markers as well as CD34 and CD117 (markers of hematopoietic stem cells), CD105 (marker of MSCs) and CD133 (marker of VSELs) (Fig. S4). Interestingly, 50% of the Lgr5+ population was CXCR4+ (Fig. S4). In conclusion, our Lgr5+ SB cells had different markers from HSCs, MSCs, VSELs and hematopoietic cells and were thus determined to be a new type of stem cell.

### 2.3 Lgr5+ SB cells are able to differentiate *in vitro*

**Figure 3:**
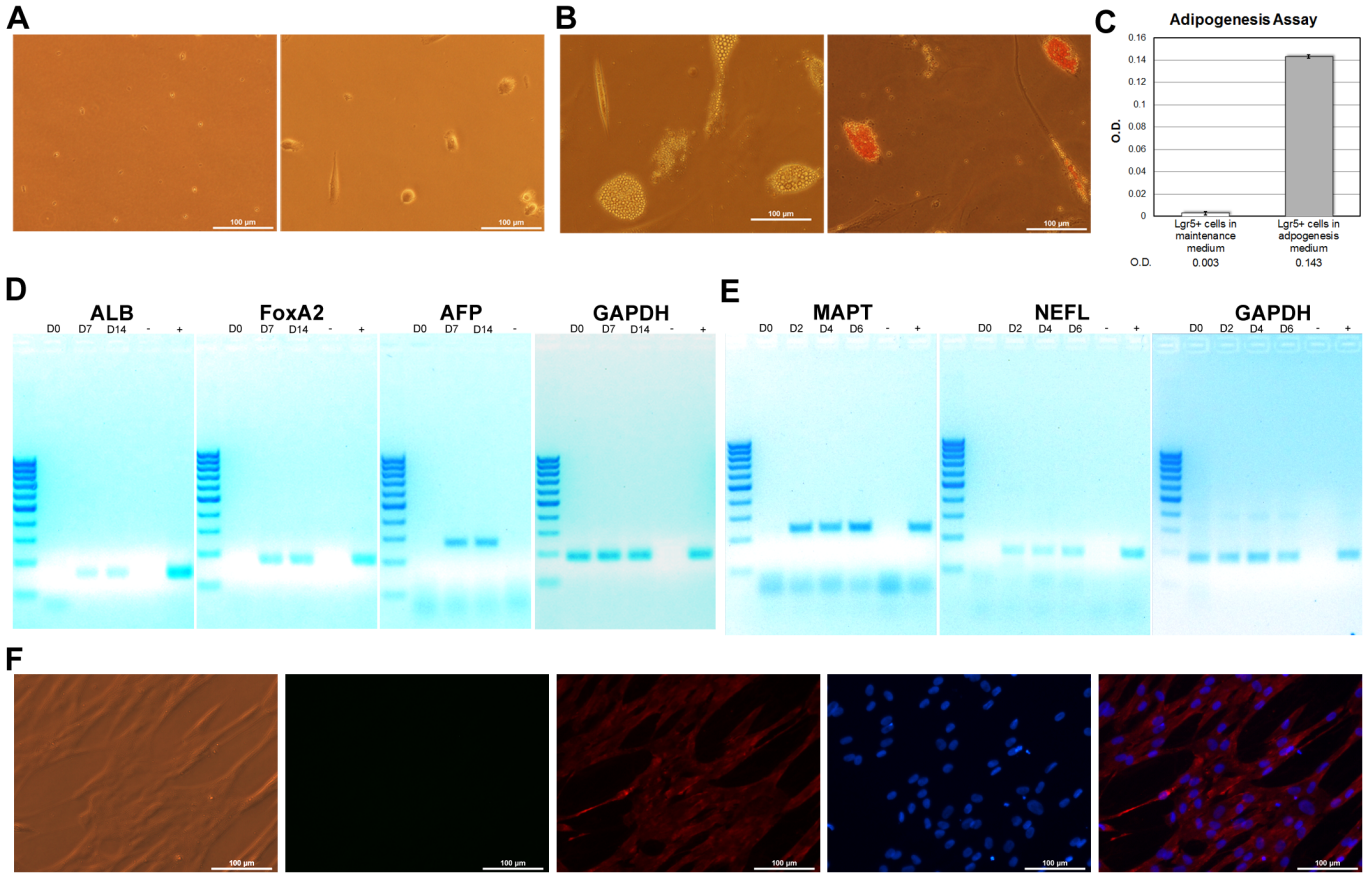
hBM-derived SB cells differentiate *in vitro*. **A) SB cell attachment.** Purified Lgr5+ cells at day 0 (left) and after attachment (right). **B) Mesoderm differentiation.** Before (left) and after (right) Oil Red O staining for differentiated adipocytes. **C) Quantification of Oil Red O- stained cells.** Negative controls: SB cells cultured in adipogenesis medium only and SB cells cultured in Stem Pro 34 medium, which is not an adipocyte differentiation media. **D) Endoderm differentiation.** RT-PCR assay for hepatocyte formation; “-” indicates the negative controls without template cDNA. Expected product sizes: GAPDH (2), 184 bp; albumin, 152 bp; FoxA2, 164 bp; and alpha-fetoprotein, 272 bp. **E) Ectoderm differentiation.** RT-PCR assay for differentiated neurons; “-” indicates negative controls without template cDNA. Expected product sizes: GAPDH (2), 184 bp; MAPT, 235 bp; and NEFL (neurofilament), 184 bp. Note: * indicates P≤0.01. **F) Immunocytochemistry using neurofilament (Cyc-3) staining.** Filters (left to right): red (Cyc-3), UV (DAPI), FITC (background), and merged Cyc-3 + DAPI. All scale bars, 100 µm.

As shown in Figure 3A, after 2 weeks of culture, the SB cells from hBM had grown to 6 –25 ìm in diameter. This proliferation and size increase suggested the presence of small stem cells.

To illustrate the SB cells' ability to differentiate into the three germ layers *in vitro*, these cells were cultured in four different types of media and subsequently analyzed to confirm that the differentiation was successful. This *in vitro* characterization was performed four times (three times using the StemCell PE isolation kit and one time using FACSorting). To test for mesoderm differentiation, the cells were cultured in adipogenesis medium. Oil Red O staining indicated significant increases in the number of adipocytes compared with the negative control (Fig. 3B, 3C). The SB cells also differentiated into hepatocytes and endoderm cells in the hepatocyte differentiation media. These cells secreted 50 ng/ml albumin into the medium and expressed several hepatocyte-specific genes such as albumin, FoxA2, and alpha-fetoprotein (Fig. 3D). To investigate the potential of ectoderm differentiation, the SB cells were similarly cultured in neuronal differentiation medium. Neurofilament and MAPT expression increased with each successive day in culture (Fig. 3E). The results from the ICC neurofilament staining also confirmed the cells' potential to neuronally differentiate (Fig. 3F). In addition to ICC, western blot analysis was also used to determine the expression of Tau protein, albumin, and adiponectin on SB cells treated with neuronal differentiation medium, hepatocyte differentiation medium, and adipocyte differentiation medium (Fig. S5). Taken together, the SB cells were able to differentiate *in vitro* into the three different germ layer cell types.

### 2.4 SB cell engraftment in SCID mice

**Figure 4:**
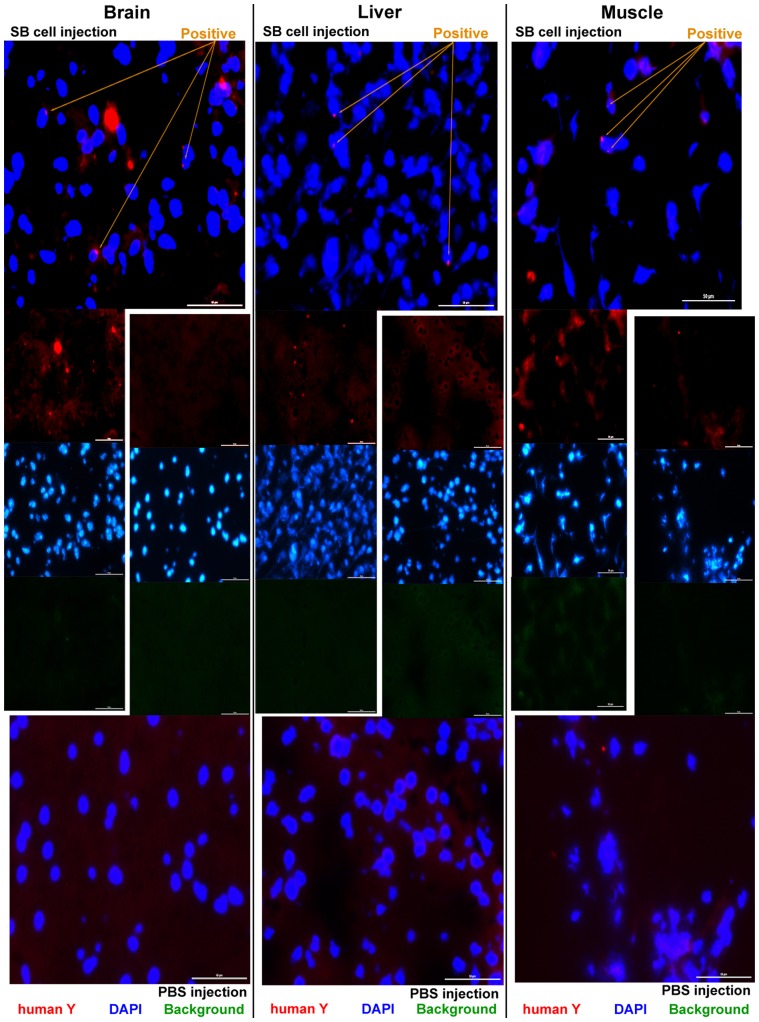
hBM-derived SB cells in a mouse xenograft model: FISH staining of mouse tissue sections. Mouse organs were collected 2 months post-injection. For each tissue, the SB cell-injected mice (top rows) were compared with the PBS injected-mice (bottom row). The human Y-chromosome Cyc-3 probe was used for FISH staining. The sections were also counterstained with DAPI. Filters: merged Cyc-3 + UV, red (Cyc-3), UV (DAPI), and FITC (background) for top row and red (Cyc-3), UV (DAPI), and FITC (background) and merged Cyc-3 + UV for bottom row. Scale bars, 50 µm.

**Figure 5:**
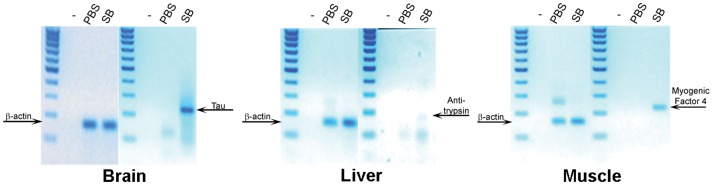
Xenografts of hBM-derived SB cell differentiation *in vivo*: RT-PCR. Mouse organs were collected 2 months post-injection. For each tissue, the SB cell-injected mice were compared with the PBS-injected mice. Marker: 100-bp DNA ladder. Expected sizes: beta-actin, 160 bp; Tau, 235 bp; α1-anti-trypsin, 175 bp; and myogenic factor 4, 225 bp.

Because SB cells can differentiate *in vitro*, they may exhibit characteristics similar to those of embryonic stem cells, which differentiate as multipotent stem cells. Thus, we performed an *in vivo* cell tracking assay. Tail-vein injections with either 1×10^5^ male SB cells from hBM or PBS were performed in sub-lethally irradiated female SCID mice. The use of SCID mice, which lack T cells and B cells, ensured that the sub-lethal irradiation would eliminate the NK cells, which have the potential to reject the SB cells in an immune system response. In addition, this type of irradiation creates an injury signal, which is important in the guiding stem cells to the injury site for repair. After 60 days, the organs of the mice were collected and analyzed. The FISH technique, which employs fluorescent human specific Y-chromosome probes, was used to detect the human cells *in vivo*. As observed in the mouse brain, liver, and muscle tissue samples, cells that were double-positive for Cyc-3 and DAPI were of human origin (Fig. 4). The 60× magnification images of the FISH analysis are found in Fig. S6. The lung, kidney, and intestine showed an increased amount of SB cells compared with the brain, which was associated with the fewest number of integrated SB cells (data not shown). These results were consistent for all six mice in the experimental group, suggesting that the injury signal guided the migration of the SB cells to the injury site. To determine whether the SB cells that migrated to the organs were capable of differentiating, RT-PCR was performed. β-actin, α1-anti-trypsin, myogenic factor 4, and Tau gene expression were assessed in these organs (Fig. 5). In this assay, primers against mouse beta-actin served as the positive control, and the primers for the liver, brain, and skeletal muscle were human-specific. The final gel analysis of the RT-PCR products demonstrated that human SB cells resided in the mouse brain, liver, and skeletal muscles and differentiated into hepatocytes, neurons, and skeletal muscle cells in the host. In addition to RT-PCR, IHC staining was also used to test the injected SB cells for differentiation in the host SCID-mice. The IHC results demonstrated that the injected SB cells, in addition to those migrating in the mouse body, also differentiated into neurons in the brain, hepatocytes in the liver, and myocytes in the muscle (Fig. S7). These data were consistent for all six mice in the experimental group.

## Discussion

The search for pluripotent or multipotent stem cells to treat degenerative diseases has been an ongoing effort. Our study makes a significant contribution to current stem cell research as we found novel multipotent stem cells in the hBM, which we refer to as SB cells. When cultured in conditioned media, these newly discovered SB cells exhibit the ability to expand and differentiate into neurons, liver cells, and muscle cells *in vitro* and *in vivo*, confirming their multipotency.

Flow cytometry and transwell experiments were used to characterize the SB cells. The cells in the SB mixture were smaller than RBCs, passed easily through a 5-µm filter and were positive for SYTO, DAPI, and Y-chromosome FISH staining. Based on this information, stem cells smaller than 6 ìm in diameter are present in hBM and hPB, which we named SB cells. Flow cytometry analyses showed that SB cells with a diameter of 1- 6 ìm expressed Lgr5, a Wnt target gene that may play a role in cell self-renewal and proliferation [36]–[38] and functions as a stem cell marker primarily in the stomach and small intestine [39]. Our study revealed that Lgr5+ cells are also found circulating in the blood and BM and may perform a profound function in all tissues of the body. The knockout of Lgr5 in mice has been shown to be lethal [40], suggesting that Lgr5 may be an early marker of epithelial progenitors and, thus, a marker of multipotency. This idea is supported by the ability of the SB cells to proliferate and differentiate. Additionally, the Lgr5 ligand is thought to be similar in sequence to the receptors of LH, FSH, TSH, and BMP, hormones that regulate homeostasis [41]. Thus, Lgr5 may participate in the maintenance of homeostasis through its circulation. The presence of the abovementioned markers suggests that SB cells have a similar phenotype to that of undifferentiated stem cells. Furthermore, the SB cells expressed Oct4 and Nanog (Fig. S3), two markers that are associated with embryonic stem cells [42]–[44] and adult MSCs [45]. The expression of these factors suggested that SB cells have the potential to differentiate. This hypothesis was tested in our *in vitro* study (Fig. 3) and *in vivo* cell-tracking assay (Fig. 4 and Fig. 5).

These SB cells were characteristically different from the previously identified small stem cells. At least three types of stem cells smaller than 6 µm have been identified in previous studies, namely, spore-like cells, blastomere-like stem cells (BLSCs), and very small embryonic-like stem cells (VSELs). Spore-like cells [26], which are present in all tissues, are composed of greater than 50% nucleic acids by volume and are protected by a glycolipid membrane. When activated under extreme conditions such as low oxygen and high or low temperatures, they can proliferate and differentiate into any cell type. However, further examination of these cells is limited by the lack of well-characterized cell markers. BLSCs, which are considered to be totipotent stem cells smaller than 5 µm [30], have been identified using the surface marker CEA (CD66e). Furthermore, VSELs are characterized by CD133 and SSEA1 expression [29]. Similar to embryonic stem cells, these cells exhibit a high ratio of nuclear volume to cytoplasmic volume and express Oct4, Nanog, and Sox2 [46].

Unlike spore-like cells, BLSCs, and VSELs, SB cells do not typically express CD66e, CD133, or Sox2, as shown in Fig. 2D. They also do not express the HSC and MSC markers CD34, CD105, and CD117. Rather, SB cells may be identified via their Lgr5 expression and positive nuclear staining with SYTO and DAPI, making them different from spore-like cells, BLSCs and VSELs. In addition, SB cells may differentiate into hepatocytes, neurons, and muscle cells *in vivo* and *in vitro*, a finding that has not yet been shown for VSELs [47], BLSCs or spore-like cells.

The use of the Lgr5 antibody to isolate Lgr5+ cells from hBM yielded a small homogenous suspension of SB cells. These cells were cultured in specific medium to obtain cells that ranged from 6 ìm to 25 ìm in diameter. Because our medium contained R-spondin-1, an agonist [48] of Wnt signaling, and the SB cells expressed Lgr5 [49], Wnt signaling may be associated with successful SB cell growth and *in vitro* expansion. Once the Lgr5+ SB cells underwent expansion, they were able to differentiate into hepatocytes, neurons, and muscle cells. Interestingly, Lgr5+ and Lgr5- proliferation assays revealed that the doubling times were similar among both cell populations (Fig. S2), contrary to our hypothesis. Further study indicated that a portion of the Lgr5- population expressed CD349 (unpublished results), a cell marker that is linked to the Wnt receptor, which leads us to suspect that Wnt signaling plays a large part in small stem cell proliferation. These data have not been previously demonstrated, which further suggests that SB cells are distinct from other small stem cell populations. Finally, Danova-Alt et al. (2012) previously dismissed the existence of small human stem cells [31], most likely due to their incorrect isolation of a VSEL population [47]. Miyanishi et al. (2013) also discovered that VSELs were not present in mice BM [50], which is similar to our result in the human SB mixture. However, VSELs may be able to originate from other human organs. Here, we provide evidence demonstrating the existence of LGR5+ small stem cells from hBM that have the potential to differentiate *in vitro* and *in vivo*.

Interestingly, SB cells were also found in hPB. The doubling time of the SB cells from hPB varied from 30∼60 hrs, depending on the person (data not shown). The ability of hPB-derived SB cells to differentiate has been demonstrated to be difficult to achieve. The disparity between SB cells from hBM and SB cells from hPB may be due to a niche difference because hBM contains many more supporting stromal cells than hPB [51]. These stromal cells comprise a basic niche environment and provide proper signaling and regulation to the surrounding stem cells for differentiation. Thus, the co-culture of stromal cells with SB cells derived from hPB may be required to form larger SB cells (data not shown). Further investigation is necessary to determine a valid cause for the proliferation and differentiation of SB cells in hPB.

As previously described, SB cells are commonly found in the dormant G0 state, and these cells transition to G1 phase only upon proper activation, which can be induced by oxygen deprivation, extreme temperatures, injury or disease. In a previous study, VSEL activation via an Ezh2-dependent bivalent domain mechanism was proposed [52]. Ezh2, a histone methyltransferase that catalyzes the trimethylation of histone H3 at lysine 27, is responsible for the gene repression of the lineage-regulatory genes for pluripotency, while the H3 lysine 4 trimethylation encodes for genes that are activated during differentiation [53]. A decrease in Ezh2 expression caused this bivalent domain to become monovalent upon activation, opening the chromatin for transcription, which enables VSEL differentiation. Successful SB cell differentiation *in vitro* and *in vivo* likely follows the same mechanism; thus, we aimed to investigate whether the activation of SB cells could be elicited in response to injury. In our *in vivo* animal model, sub-lethal gamma-irradiation was used to provide the injury signal necessary for the mobilization of human SB cells throughout the mouse bodies into organs such as the brain, muscle, and liver in a similar fashion to that of VSELs, which have been shown to mobilize only to the site of craniotomy to form skeletal tissue [54]. Such an injury signal may be critical for the activation, expansion, and differentiation of SB cells. This injury signal may also account for the existence of a very heterogeneous population of small stem cells (spore-like, BLSCs, VSELs, and SB cells). The degree of the injury or different types of stress signals may call for different responses that require specific cells. However, how this injury signal is associated with stem cell activation through pathways such as the Ezh2-dependent bivalent domain remains unclear and requires further investigation.

In the future, we hope to study this G0 to G1 activation by focusing our efforts on the SB cells post-activation. We propose that mobile SB cells from a particular patient could undergo *in vivo* expansion and enrichment *in vitro* prior to injection into an injured patient. This *in vivo* method would preserve any SB cell characteristics and chromosome epigenetics that may be altered using traditional *in vitro* culture. If this clinical trial proves to be successful, our SB cells may hold great promise for future cell therapy because adult stem cells do not induce autologous cell teratoma formation (unpublished result) or immune rejection [55] nor do they raise any ethical concerns.

## Conclusion

In summary, Lgr5-expressing SB cells isolated from human bone marrow can expand *in vitro* in culture and differentiate both *in vitro* and *in vivo*.

## Supporting Information

Figure S1 **Quantitation of DAPI+ Lgr5+ cells.** DAPI staining of the Lgr5+ cells obtained from the bead separation protocol revealed that 70% of these cells were DAPI+.
(TIF)

Figure S2 **A) Proliferation rate of Lgr5+ and Lgr5- cells in the BM.** The BM supernatant was collected and purified using magnetic beads to select the Lgr5+ cells. The proliferation rates of both the Lgr5+ and Lgr5- cells were determined. The purified Lgr5+ cells and Lgr5- cells were plated in triplicate for each time point in 48-well plates at 3×10^4^ cells/well. For each well, 200 µl medium was added. The total amount of cells in each well was determined using a hemocytometer at 0 hrs, 24 hrs, 48 hrs, and 96 hrs of incubation. The proliferation rate of the Lgr5+ cells was 21.7 hrs (left), and the rate of Lgr5- cells was 18.8 hrs (right). **B) Cell-cycle profile analysis of the SB cells.** The DNA content in the purified Lgr5+ SB cells was analyzed by flow cytometry analysis in which the number of cells and the amount of DNA content were compared after 0 hrs, 8 hrs, and 14 hrs. The percentage of cells in each phase of the cell cycle was calculated using the ModFit software.
(TIF)

Figure S3 **Gene expression in the SB cells.** RT-PCR analysis indicated that the SB cells expressed GAPDH and the embryonic stem cell markers Oct4 and Nanog. Expected sizes: GAPDH (1), 296 bp; Oct4, 225 bp; Nanog, 190 bp; and Sox2, 159 bp.
(TIF)

Figure S4 **Marker exression in Lgr5+ cells.** Flow cytometry was applied to determine the expression of Lin, CD45, CD117, CD34, CD105, CD133, and CXCR4 in the Lgr5+ cells from the SB mixture derived from hBM. The data showed that the Lgr5+ cells were Lin-, CD345-, CD34-, CD105-, CD117-, and CD133-. In addition, 50% of the Lgr5+ cell population was positive for CXCR4 expression.
(TIF)

Figure S5 ***In vitro*** **differentiation of SB cells.** From left to right, the SB cells were treated with neuron differentiation medium, hepatocyte differentiation medium, and adipocyte differentiation medium, respectively. β-actin was used as a positive protein existence control for all samples. For the neuron differentiation medium-treated cells (right), Tau and β-actin expression were tested at 0 days and 8 days of incubation. The hepatocyte differentiation medium-treated cells (middle) were collected at 0 days and 7 days of incubation to test for the expression of albumin and β-actin. The cells cultured in adipocyte differentiation medium (left) were lysed to test for adiponectin and β-actin at 0 days and 20 days.
(TIF)

Figure S6 **Xenograft SB cell migration** ***in vivo*** **: 60X images of frozen mouse tissue sections subjected to FISH staining.** Fig. 4 illustrated the FISH staining of frozen mouse tissue sections at 40X. Mouse organs were collected two months post-injection. The human Y-chromosome Cyc-3 probe (red) was used for FISH staining. The sections were counterstained with DAPI. The organs collected were the brain, liver, and muscle (left to right). Filters (top to bottom): UV (DAPI), red (Cyc-3), FITC (background), and merged Cyc-3 + UV. Scale bars, 20 µm (DAPI, red, FITC) and 10 µm (merge).
(TIF)

Figure S7 **Xenograft SB cell differentiation** ***in vivo*** **. IHC staining of post-injection tissues.** Organs were collected two months post-injection. For each tissue, the SB cell-injected mice (top rows) were compared with the PBS injected-mice (bottom row). Filters (left to right): red (Cyc-3), FITC (background), UV (DAPI), and merged Cyc-3 + UV. ***Brain*** **:** Staining for neurofilaments (human-specific) and DAPI. T ***Liver*** **:** Staining for *alpha*-1-antitrypsin (human-specific) and DAPI. ***Muscle*** **:** Staining for dystrophin (human-specific) and DAPI.
(TIF)
