# Supplementary figures and images for: Identification of a Distinct Small Cell Population from Human Bone Marrow Reveals Its Multipotency In Vivo and In Vitro
Source: PLoS One. 2014 Jan 17;9(1):e85112. doi: 10.1371/journal.pone.0085112 (PMC3894949; doi:10.1371/journal.pone.0085112)

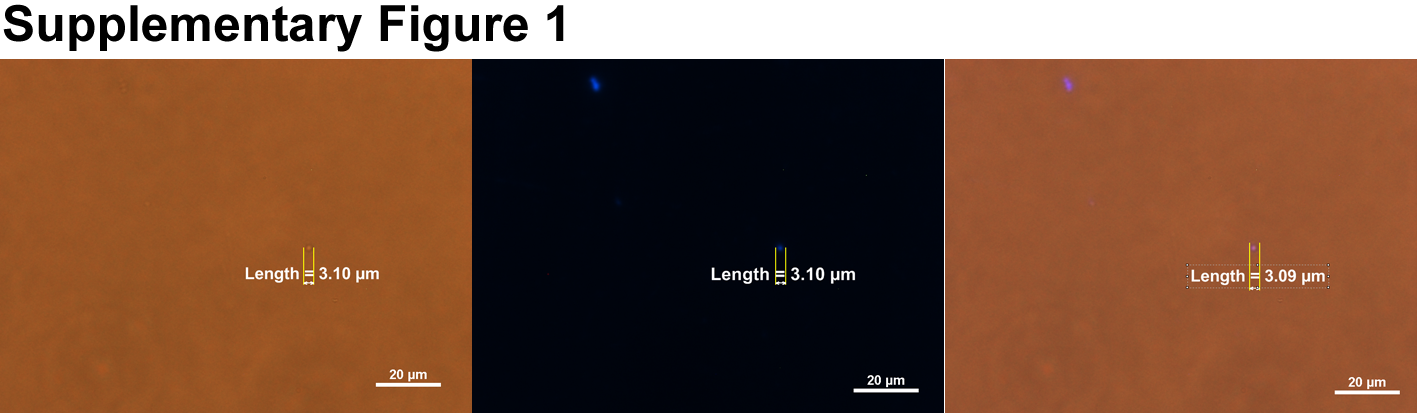

Supplement: Figure S1 — Quantitation of DAPI+ Lgr5+ cells. DAPI staining of the Lgr5+ cells obtained from the bead separation protocol revealed that 70% of these cells were DAPI+. (TIF) [file pone.0085112.s001.tif]

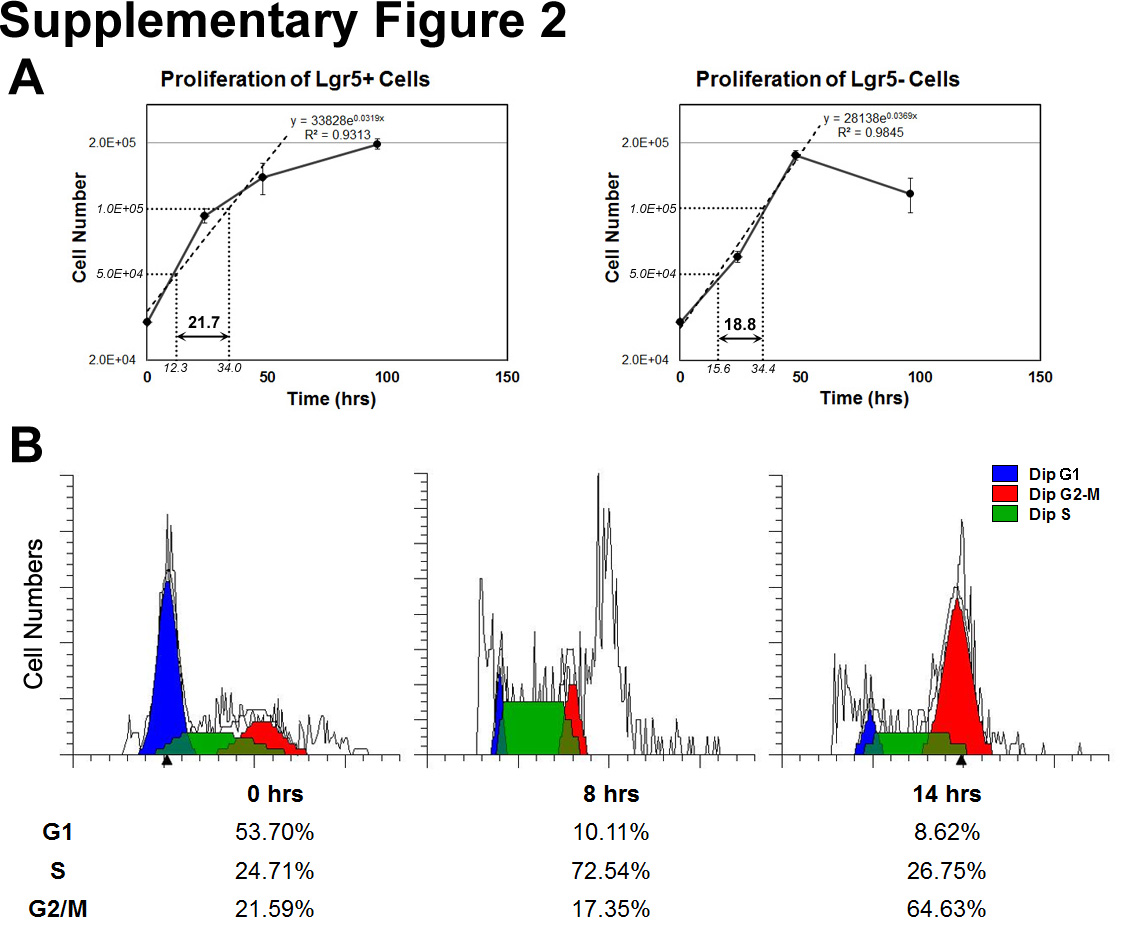

Supplement: Figure S2 — A) Proliferation rate of Lgr5+ and Lgr5- cells in the BM. The BM supernatant was collected and purified using magnetic beads to select the Lgr5+ cells. The proliferation rates of both the Lgr5+ and Lgr5- cells were determined. The purified Lgr5+ cells and Lgr5- cells were plated in triplicate for each time point in 48-well plates at 3×10^4^ cells/well. For each well, 200 µl medium was added. The total amount of cells in each well was determined using a hemocytometer at 0 hrs, 24 hrs, 48 hrs, and 96 hrs of incubation. The proliferation rate of the Lgr5+ cells was 21.7 hrs (left), and the rate of Lgr5- cells was 18.8 hrs (right). B) Cell-cycle profile analysis of the SB cells. The DNA content in the purified Lgr5+ SB cells was analyzed by flow cytometry analysis in which the number of cells and the amount of DNA content were compared after 0 hrs, 8 hrs, and 14 hrs. The percentage of cells in each phase of the cell cycle was calculated using the ModFit software. (TIF) [file pone.0085112.s002.tif]

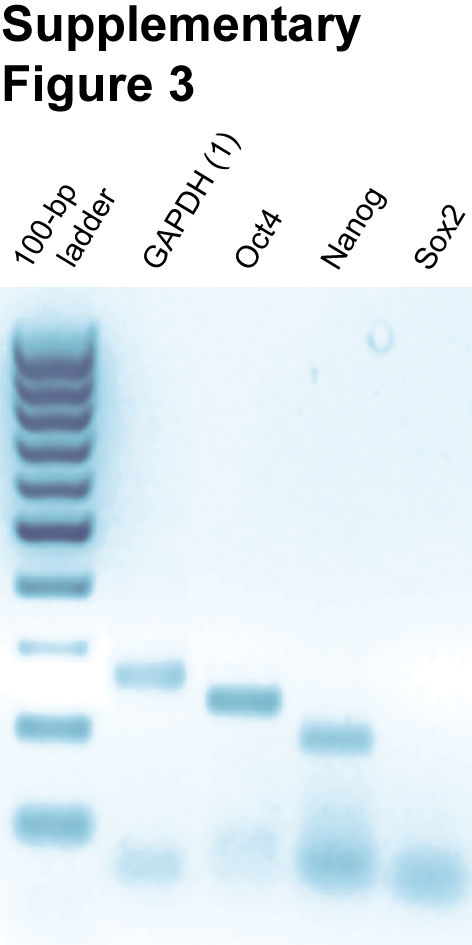

Supplement: Figure S3 — Gene expression in the SB cells. RT-PCR analysis indicated that the SB cells expressed GAPDH and the embryonic stem cell markers Oct4 and Nanog. Expected sizes: GAPDH (1), 296 bp; Oct4, 225 bp; Nanog, 190 bp; and Sox2, 159 bp. (TIF) [file pone.0085112.s003.tif]

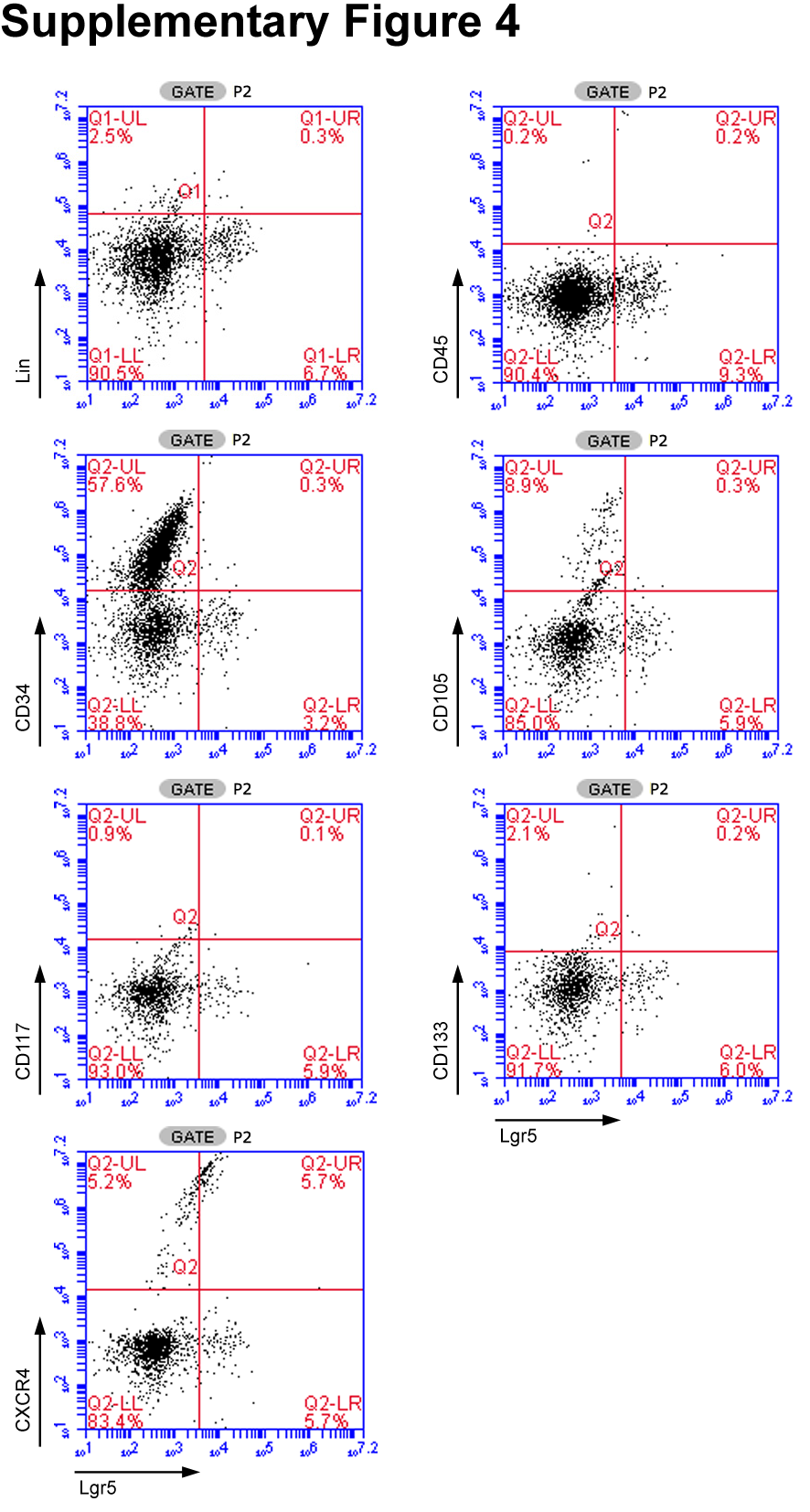

Supplement: Figure S4 — Marker exression in Lgr5+ cells. Flow cytometry was applied to determine the expression of Lin, CD45, CD117, CD34, CD105, CD133, and CXCR4 in the Lgr5+ cells from the SB mixture derived from hBM. The data showed that the Lgr5+ cells were Lin-, CD345-, CD34-, CD105-, CD117-, and CD133-. In addition, 50% of the Lgr5+ cell population was positive for CXCR4 expression. (TIF) [file pone.0085112.s004.tif]

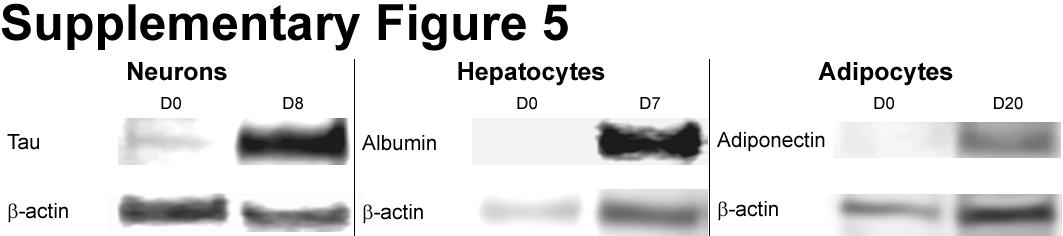

Supplement: Figure S5 — In vitro differentiation of SB cells. From left to right, the SB cells were treated with neuron differentiation medium, hepatocyte differentiation medium, and adipocyte differentiation medium, respectively. β-actin was used as a positive protein existence control for all samples. For the neuron differentiation medium-treated cells (right), Tau and β-actin expression were tested at 0 days and 8 days of incubation. The hepatocyte differentiation medium-treated cells (middle) were collected at 0 days and 7 days of incubation to test for the expression of albumin and β-actin. The cells cultured in adipocyte differentiation medium (left) were lysed to test for adiponectin and β-actin at 0 days and 20 days. (TIF) [file pone.0085112.s005.tif]

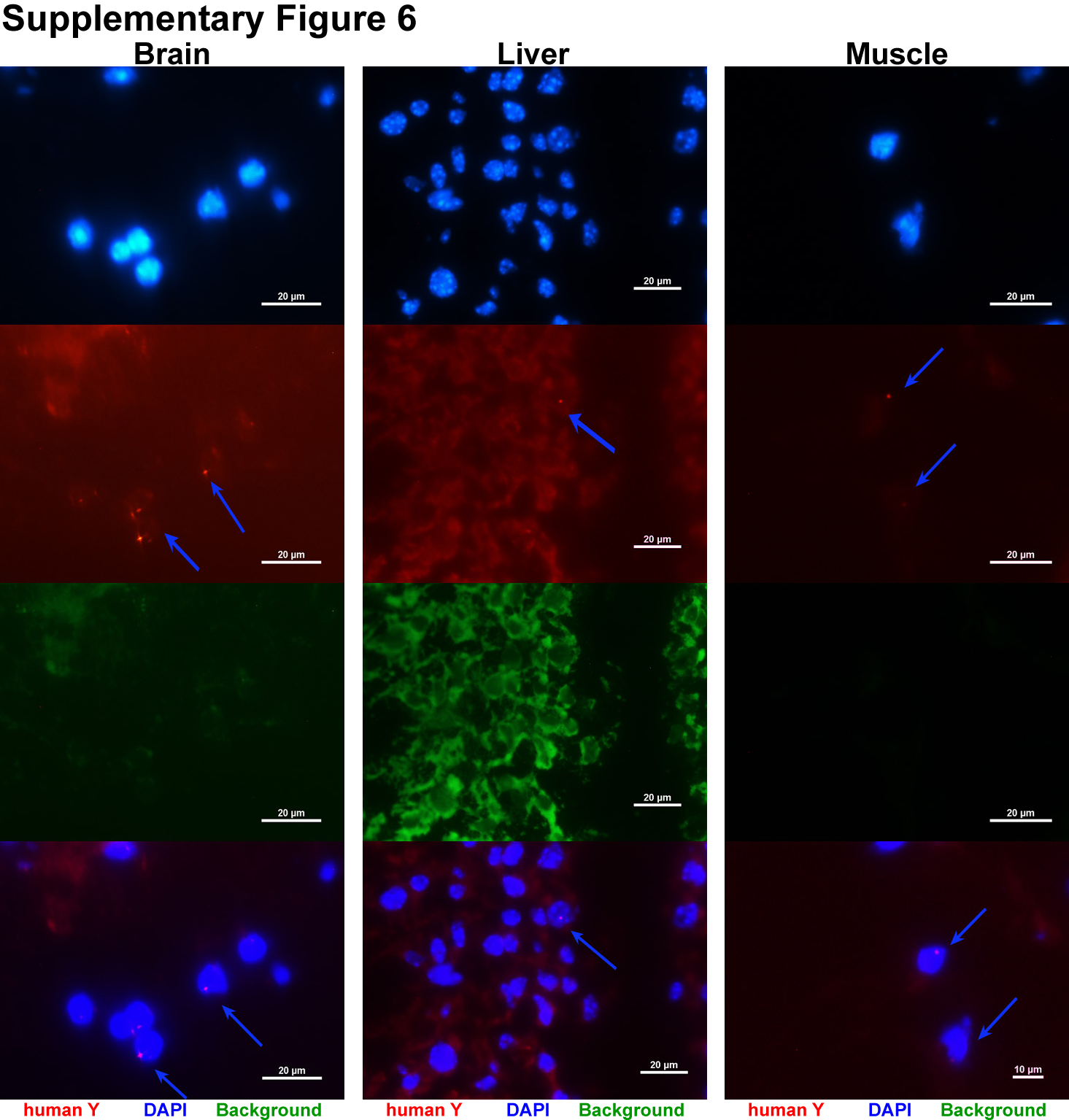

Supplement: Figure S6 — Xenograft SB cell migration in vivo : 60X images of frozen mouse tissue sections subjected to FISH staining. Fig. 4 illustrated the FISH staining of frozen mouse tissue sections at 40X. Mouse organs were collected two months post-injection. The human Y-chromosome Cyc-3 probe (red) was used for FISH staining. The sections were counterstained with DAPI. The organs collected were the brain, liver, and muscle (left to right). Filters (top to bottom): UV (DAPI), red (Cyc-3), FITC (background), and merged Cyc-3 + UV. Scale bars, 20 µm (DAPI, red, FITC) and 10 µm (merge). (TIF) [file pone.0085112.s006.tif]

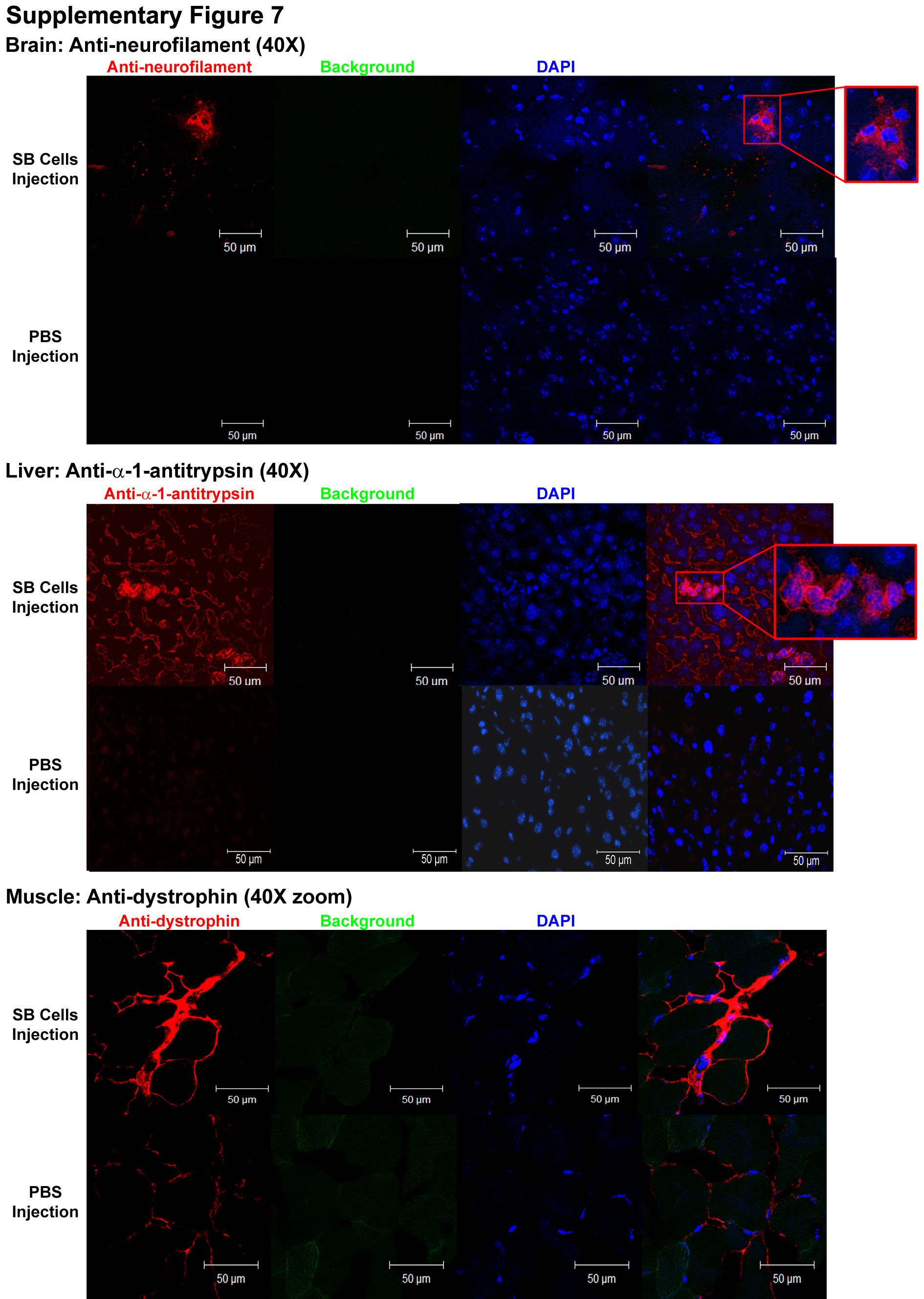

Supplement: Figure S7 — Xenograft SB cell differentiation in vivo . IHC staining of post-injection tissues. Organs were collected two months post-injection. For each tissue, the SB cell-injected mice (top rows) were compared with the PBS injected-mice (bottom row). Filters (left to right): red (Cyc-3), FITC (background), UV (DAPI), and merged Cyc-3 + UV. Brain : Staining for neurofilaments (human-specific) and DAPI. T Liver : Staining for alpha-1-antitrypsin (human-specific) and DAPI. Muscle : Staining for dystrophin (human-specific) and DAPI. (TIF) [file pone.0085112.s007.tif]
